# Supplementary material for: Challenges of conducting an international observational study to assess immunogenicity of multiple COVID-19 vaccines
Source: PLOS Glob Public Health. 2023 Jun 20;3(6):e0001918. doi: 10.1371/journal.pgph.0001918 (PMC10281570; doi:10.1371/journal.pgph.0001918)
Supplement: Attachment — Submitted filename: Response_to_Reviewers_Challenges_Manuscript 04142023.docx [file pgph.0001918.s001.docx]

April 14, 2023

Dear Dr. Asweto,

Thank you for the opportunity to submit our revised manuscript. We appreciate you and the reviewers taking the time to read our paper and provide insightful comments. We have considered your feedback and incorporated your suggested changes into the current revised version.

Below are our point-by-point responses. We hope we have addressed all your questions and that the revised version meets your high standards. We welcome any additional comments. A marked copy of the original manuscript as well as a clean copy without track changes has been uploaded as per the instructions received.

Please note that we have revised the author list to include Sally Hunsberger, InVITE’s principal investigator, who was inadvertently left off. We have also changed Placide Mbala Kingebeni from second author to co-first author.

PGPH-D-23-00316
Challenges of conducting an international observational study to assess immunogenicity of multiple COVID-19 vaccines
PLOS Global Public Health

Point-by-point responses to reviewers’ comments:

*1. Please amend your detailed online Financial Disclosure statement. This is published with the article. It must therefore be completed in full sentences and contain the exact wording you wish to be published.*

*a) State the initials, alongside each funding source, of each author to receive each grant.*

Response: The following language has been added to the Financial Disclosure statement for clarity: “This research was supported by the Intramural Research Program of the NIAID/NIH; no grant money was received from any outside source.”

*b) State what role the funders took in the study. If the funders had no role in your study, please state: “The funders had no role in study design, data collection and analysis, decision to publish, or preparation of the manuscript.”*

Response: The Financial Disclosure statement has been revised and now states “This project has been funded in whole or in part with federal funds from the National Cancer Institute, National Institutes of Health, under Contract No. 75N91019D00024. The content of this publication does not necessarily reflect the views or policies of the Department of Health and Human Services, nor does mention of trade names, commercial products, or organizations imply endorsement by the U.S. Government. This research was supported by the Intramural Research Program of the NIAID/NIH; no grant money was received from any outside source. The funders collaborated with the participating countries on study design, data collection and analysis, decision to publish, and preparation of the manuscript.”

*2. In the Funding Information you indicated that no funding was received. Please revise the Funding Information field to reflect funding received.*

*Please ensure that the funders and grant numbers match between the Financial Disclosure field and the Funding Information tab in your submission form. Note that the funders must be provided in the same order in both places as well.*

Response: The Funding Information has been revised to show that the study was funded by NIH/NIAID. No grant money was received from any outside source.

*3. Please provide separate main figure files in .tif or .eps format only and ensure that all files are under our size limit of 10MB.*

*For more information about how to convert your figure files please see our guidelines:* [*https://journals.plos.org/globalpublichealth/s/figures*](https://journals.plos.org/globalpublichealth/s/figures)

Response: The figure files have been formatted as suggested in the revised submission.

*4. Please review your reference list to ensure that it is complete and correct. If you have cited papers that have been retracted, please include the rationale for doing so in the manuscript text or remove these references and replace them with relevant current references. Any changes to the reference list should be mentioned in the rebuttal letter that accompanies your revised manuscript. If you need to cite a retracted article, indicate the article’s retracted status in the References list and also include a citation and full reference for the retraction notice.*

Response: No references have been retracted. The reference list has been reviewed for accuracy and completeness. Many thanks.

Reviewers' comments:

Reviewer's Responses to Questions

Comments to the Author

*1. Does this manuscript meet PLOS Global Public Health’s* [*publication criteria*](https://gcc02.safelinks.protection.outlook.com/?url=https%3A%2F%2Fjournals.plos.org%2Fglobalpublichealth%2Fs%2Fcriteria-for-publication&data=05%7C01%7Crsardana%40niaid.nih.gov%7C2ae9aeaf384c4af0651b08db2be0aa44%7C14b77578977342d58507251ca2dc2b06%7C0%7C0%7C638152016144676512%7CUnknown%7CTWFpbGZsb3d8eyJWIjoiMC4wLjAwMDAiLCJQIjoiV2luMzIiLCJBTiI6Ik1haWwiLCJXVCI6Mn0%3D%7C3000%7C%7C%7C&sdata=D%2Ft%2Byq6w9PdDWvHz6i%2Bg7aJc5t1BhmuYu1xxdm9Ef50%3D&reserved=0)*? Is the manuscript technically sound, and do the data support the conclusions? The manuscript must describe methodologically and ethically rigorous research with conclusions that are appropriately drawn based on the data presented.*

*Reviewer #1: Partly*

*Reviewer #2: Yes*

Response: Many thanks for your review.

*2. Has the statistical analysis been performed appropriately and rigorously?*

*Reviewer #1: Yes*

*Reviewer #2: N/A*

Response: Your review is much appreciated.

*3. Have the authors made all data underlying the findings in their manuscript fully available (please refer to the Data Availability Statement at the start of the manuscript PDF file)?

The* [*PLOS Data policy*](https://gcc02.safelinks.protection.outlook.com/?url=https%3A%2F%2Fjournals.plos.org%2Fglobalpublichealth%2Fs%2Fdata-availability&data=05%7C01%7Crsardana%40niaid.nih.gov%7C2ae9aeaf384c4af0651b08db2be0aa44%7C14b77578977342d58507251ca2dc2b06%7C0%7C0%7C638152016144676512%7CUnknown%7CTWFpbGZsb3d8eyJWIjoiMC4wLjAwMDAiLCJQIjoiV2luMzIiLCJBTiI6Ik1haWwiLCJXVCI6Mn0%3D%7C3000%7C%7C%7C&sdata=%2BXYyY6UhMUxhYmDPULYg5C%2FTYarP87t6BBnvuCmSn1g%3D&reserved=0) *requires authors to make all data underlying the findings described in their manuscript fully available without restriction, with rare exception. The data should be provided as part of the manuscript or its supporting information or deposited to a public repository. For example, in addition to summary statistics, the data points behind means, medians and variance measures should be available. If there are restrictions on publicly sharing data—e.g., participant privacy or use of data from a third party—those must be specified.*

*Reviewer #1: Yes*

*Reviewer #2: Yes*

Response: Many thanks!

*4. Is the manuscript presented in an intelligible fashion and written in standard English?

PLOS Global Public Health does not copyedit accepted manuscripts, so the language in submitted articles must be clear, correct, and unambiguous. Any typographical or grammatical errors should be corrected at revision, so please note any specific errors here.*

*Reviewer #1: Yes*

*Reviewer #2: Yes*

Response: Many thanks!

*5. Review Comments to the Author*

*Comment: The only data presented (graphically) is the submission of enrollees by country and date. The number and demographics of enrollees by country was not presented.*

Response: The focus of this paper was to describe the challenges we faced and the solutions we implemented so that other studies facing similar challenges can benefit from our lessons learned. The rapid design, initiation, and enrollment of the study (enrollment targets for each country have been met) demonstrate that the challenges were successfully mitigated. The original manuscript stated in lines 75-77 “With nearly 5,500 adults enrolled, the study is prospectively following participants for approximately two years, a period longer than many other vaccine immunogenicity studies.” We have achieved our enrollment target and revised the manuscript to: “A total of 5,401 adults have been enrolled, and the study is prospectively following participants for approximately two years, a period longer than many other vaccine immunogenicity studies.”

*Comment: This paper does not address the entirety of its topic. The aim of this paper as stated was to evaluate/assess immunogenicity, reactogenicity and efficiency of COVID-19 vaccine among the selected countries through an observational study, however these key issues were not addressed in the write up. Hence the paper does not speak to the Topic.
There is need to bring in the immunogenicity, reactogenicity and the efficiency of COVID-19 vaccines as it relates to the enrollees in this study, as this will add more to the body of knowledge regarding the complexities surrounding COVID-19 vaccination more especially in low- and middle-income countries.
To retain this selected topic, there is need to present finding around the 3 key issues mentioned above or the Topic can be modified to focus on Challenges of COVID-19 vaccinations and not immunogenicity.*

Response: The objective of the study is to evaluate and assess the immunogenicity of COVID-19 vaccines among countries participating in InVITE. The focus of this paper is to describe the challenges that we faced and the solutions that we implemented so that other studies, especially those in low- and middle-income countries that face similar challenges, can benefit from our lessons learned.

*Reviewer #2: Sharing of lessons learnt is encouraged to support similar studies in the future, making future studies time and cost-efficient.*

Response: Thank you!

*Comment: Line 56,97 and others: The full stop is before the brackets. Check throughout the document for this issue.*

Response: This has been corrected throughout the manuscript. Thank you for noting this.

*Comment: Line 203: Introduce who community health workers are. This cadre is not available in every country or is known using a different terminology therefore, readers may not be familiar.*

Response: Line 203 of the original manuscript “Community health workers were recruited to make phone calls and home visits to remind participants of follow-up visits” has been revised accordingly in line 210 of the updated clean version of the manuscript to “Community health workers, who are members of the community, were trained to provide informal counseling, perform outreach efforts, and serve as study advocates. They were engaged as part of the study team to make phone calls and home visits to remind participants of follow-up visits.” 

*Comment: Line 248: It just says SOP- Standard Operating Procedures. The other columns for challenges and solutions are not filled.*

Response: Standard Operating Procedures was an abbreviation used in the table. We are no longer using the abbreviation in the table, and this has been removed from the revised version.

*Comment: Line 256 – 257: Speaks about capacity gained. Describe the capacity gained.*

Response: Line 256–257 of the original manuscript “The capacity gained will contribute to stronger pandemic preparedness for the future” has been revised to “This experience with study planning, regulatory submission, training, and study initiation and implementation during an ongoing pandemic has contributed to research capacity and pandemic preparedness at each of the study sites.”

*Comment: Table 1: In the study logistics column, it says that the challenge is “Study did not provide vaccines”. The study is an observational study meaning there is no intervention therefore, it is not expected that there would be the provision of vaccines by the project.*

Response: While the study did not provide vaccines, it was a logistical challenge for the study team to adjust participant visit schedules based on vaccine distributions by the local health authorities. As this was not something the study team could control or influence, close coordination with vaccination programs was required to track distribution timelines. Thus, we have included this as a challenge encountered during the implementation of the study.

*Comment: Table 1: Study logistics column “Variation in country-specific needs and requirements”. This is not clear. Does it mean in terms of ethics/research permits or that implementation had to be tailored to country-specific needs? Link to the wording in the paragraph on study logistics in lines 109-114.*

Response: Wording in row 2 of Table 1 on “Variation in country-specific needs and requirements” has been linked to the paragraph on study logistics referenced in the comment and has been revised from “Site-specific appendix to the main protocol developed for each country” to “Each country submitted a site-specific appendix to the main protocol for regulatory approval to address and fulfill their site-specific regulatory policies and procedures”.

• General comments:
o *It is useful for readers of these types of manuscripts to learn detail on how sites gained capacity during the conduct of this study. It provides insight to the usefulness of these studies and shows the preparedness of these sites to attract future studies.*

Response: We have attempted to highlight the usefulness of our study regarding capacity building by adding the following language (lines 267-270 in the clean, revised version): “This experience with study planning, regulatory submission, training, and study initiation and implementation during an ongoing pandemic has contributed to research capacity and pandemic preparedness at each of the study sites”—as well as in lines 293-298 in the revised clean manuscript: “This study serves as an example of how established programs in resource-limited settings can be leveraged to contribute to biomedical research during a pandemic response. Lessons learned from this study can be applied to other studies mounted to respond rapidly during a global health crisis and will contribute to capacity for stronger pandemic preparedness in the future when there is a crucial need for urgent response and data collection.”

*Comment: The narrative is well written but it is missing specific examples that would be useful to the reader. Example lines 182-184 “Flexibility was also required to accommodate country-specific projected vaccine delivery and distribution timelines, informed consent requirements and standard operating procedures”. What is meant by flexibility? Perhaps also giving an example of a specific country on how it was worded. Lines 205-207 are a good example of a specific description of how a specific challenge was accommodated.*

Response: Thank you for your suggestion. An example has been added to lines 182-184 referenced in the comment to address what is meant by flexibility. We have revised the manuscript accordingly: “Flexibility was also required to accommodate country-specific projected vaccine delivery and distribution timelines, informed consent requirements and standard operating procedures. For example, in Mongolia, government policy initially declared that a single booster dose was to be administered; however, this policy was updated to include receipt of a second booster dose, and the flexibility in the protocol to accommodate this allowed the enrollment of those receiving 1st and 2nd booster doses of vaccine. A second example is that recruitment needed to be tailored to the setting(s) in each country where vaccines were delivered because the local vaccination programs administered vaccines in a variety of locations, including hospitals, clinics, and mobile vans.”.

*Comment: Apart from cultural beliefs, were any challenges faced with observation of religious events or seasonality such as rains? How were these challenges overcome?*

Response: Thank you so much for the comments. There were challenges that we encountered with follow-up visits due to religious events. We have revised lines 209-214 (in the original manuscript) “To avoid missed visits, the window periods for each visit are wide (1-2 months). Despite reminders, some participants were unable to return for visits within the study windows. To maximize specimen and data collection, an SOP was developed to allow for out-of-window visits emphasizing the importance of collecting key visit samples outside of the original 1–2-month window while remaining within the protocol requirements.” The manuscript now states on lines 218-225 of the revised clean manuscript to “There were situations that made it difficult for participants to attend follow-up visits such as extended religious holidays, including Ramadan and the Lunar New Year, or travel outside of a study area due to military duty or employment. To avoid missed visits, the window periods for each visit are wide (1-2 months). Despite reminders, some participants were unable to return for visits within the study windows. To maximize specimen and data collection, an SOP was developed to allow for out-of-window visits emphasizing the importance of collecting visit samples outside of the original 1–2-month window while remaining within the protocol requirements.”

*Comment: When was the study conducted?*

Response: The study is still ongoing. We have addressed the comment in line 65 of the clean, revised version of the manuscript as follows: “The International Study on COVID-19 Vaccines to Assess Immunogenicity, Reactogenicity, and Efficacy (InVITE) was launched in August of 2021.” has been revised to “The International Study on COVID-19 Vaccines to Assess Immunogenicity, Reactogenicity, and Efficacy (InVITE) was launched in August of 2021 and is still ongoing.”

*Comment: During the period of the project were any of the countries under lockdown, curfews or restricted movement? If yes, how were these challenges overcome for follow-up?*

Response: The study was launched in August 2021 when vaccines were being administered within all participating countries. We have not had challenges related to lockdowns, curfews, or restriction in movement during the study thus far.
